# Evaluation of Sources and Patterns of Elemental Composition of PM_2.5_ at Three Low-Income Neighborhood Schools and Residences in Quito, Ecuador

**DOI:** 10.3390/ijerph14070674

**Published:** 2017-06-23

**Authors:** Amit U. Raysoni, Rodrigo X. Armijos, M. Margaret Weigel, Patricia Echanique, Marcia Racines, Nicholas E. Pingitore, Wen-Whai Li

**Affiliations:** 1 Department of Public Health Sciences, The University of Texas at El Paso, El Paso, TX 79968, USA; amit.raysoni@gmail.com; 2 Department of Environmental Health, School of Public Health, Indiana University, Bloomington, IN 47405, USA; weigelm@iu.edu; 3 Proyecto Prometeo, Secretaria de Educacion Superior, Ciencia y Tecnologia (SENESCYT), Quito, EC 170526, Ecuador; 4 Instituto de Investigaciones en Biomedicina, Universidad Central del Ecuador, Quito, EC 170201, Ecuador; mracinesorbe@hotmail.com; 5 Facultad de Medicina, Universidad Central del Ecuador, Quito, EC 170136, Ecuador; pechanique@gmail.com; 6 Department of Geological Sciences, University of Texas at El Paso, El Paso, TX 79968, USA; npingitore@utep.edu; 7 Department of Civil Engineering, University of Texas at El Paso, El Paso, TX 79968, USA; wli@utep.edu

**Keywords:** elemental composition, PM_2.5_, enrichment factors, principal component analysis, schools, residences, Quito

## Abstract

Elemental characterization of fine particulate matter was undertaken at schools and residences in three low income neighborhoods in Quito, Ecuador. The three zones were located in the northern (Cotocollao), south central (El Camal), and south east (Los Chillos) neighborhoods and were classified as zones 1–3, respectively. Forty elements were quantified via ICP-MS analysis. Amongst the geogenic elements, the concentration of Si was the most abundant followed by S, Al, and Ca. Elements with predominantly anthropogenic sources such as Zn, V, and Ni were higher in zone 3 school followed by zone 2 and zone 1 schools. Enrichment factors were calculated to study the role of crustal sources in the elemental concentrations. Geogenic elements, except K, all had values <10 and anthropogenic elements such as Ni, V, Zn, Pb, As, Cr had >10. Principal Component Analysis suggested that Ni and V concentrations were strongly attributable to pet coke and heavy oil combustion. Strong associations between As and Pb could be attributed to traffic and other industrial emissions. Resuspended dust, soil erosion, vehicular emissions (tailpipe, brake and tire wear, and engine abrasion), pet coke, heavy oil combustion, and heavy industrial operations were major contributors to air pollution.

## 1. Introduction

Air pollution in urban environments is a major cause of health concern, especially for sensitive populations like young school children. Fine particulate matter (PM_2.5_), i.e., particles less than 2.5 µm in aerodynamic diameter, is a major component of urban air pollution and has the potential to reach the small airways and alveoli-gas exchange portion of the lungs. PM_2.5_ is a complex and heterogeneous mix of constituents comprising of solid and liquid phase aerosols suspended in air [1], exhibiting a wide range of physiochemical properties and considerable heterogeneity in an urban air shed. The physical and chemical properties and biological composition of PM_2.5_ generally reflect the contributing sources at local and regional levels [2]. Many air pollution epidemiologic studies have studied the associations between PM_2.5_ mass and health effects such as respiratory morbidity [3,4,5], asthma exacerbation [6], cardiovascular health effects [7,8], and emergency room visits [9]. However, PM_2.5_ mass may not be an accurate marker for the various adverse health associations mentioned above due to the diverse mix of the PM composition. Recent studies have documented robust relationships between the toxicological effects of the metals adsorbed on PM_2.5_ particles and adverse health effects [10,11,12,13], thereby, suggesting that PM_2.5_ composition may be one of the better predictor variables associated with adverse health endpoints than PM_2.5_ mass. Other possible predictor variables could be polycyclic aromatic hydrocarbons [14], elemental carbon, and ultrafine particles. Heavy metals such as As, Cd, Co, Cu, Cr, Mn, Ni, Pb, V, Zn are known to be toxic, carcinogenic, or mutagenic [15]. Results compiled from extensive epidemiologic, toxicological, and experimental studies in the last few decades have confirmed that certain heavy metals are likely to cause cancer in animal models and human beings [16,17]. Heavy metals can be inhaled, ingested or absorbed via the dermal contact into the human body resulting in a plethora of health complications [18]. Heavy metals such as As, Cd, Cr, and Pb can lead to DNA damage due to their potential to form reactive oxygen species (ROS) [19,20,21] which could cause severe oxidative stress within cells and result in their ultimate damage due to the formation of oxidized cellular macromolecules [22,23,24].

High exposure to Cu has been linked to Wilson disease in human due to cellular damage [25]. Cakmak and colleagues documented statistically significant association between Cd, Zn, Pb, and acute changes in the cardiovascular and respiratory physiology of a cohort of young college students near a steel production plant in Canada [19]. Findings from an American Cancer Society (ACS) study suggests a plausible association between lung cancer and long term PM_2.5_ chemical carcinogens like As, Ni, and Cr [26]. A study conducted in Spokane, WA from 1995 to 2002 demonstrated a statistically significant association between ED visits for asthma and fine particulate Zn [27]. Another epidemiological study conducted on boiler makers showed a significant alteration in cardiac autonomic function and occupational metallic PM_2.5_ exposure especially lead and vanadium [28]. 

Air pollution in rapidly developing cities of many Latin American nations has garnered the attention of the public and government officials in the last few years [29,30,31]. For example, Quito, the high altitude capital city of Ecuador is plagued with severe air pollution episodes throughout the year [32]. Located at 2850 m above the sea level, this city is nestled in the Guayllabamba river basin valley between the eastern and western chains of the Andes Mountains. Inefficient combustion due to 27% lower oxygen levels at this high altitude results in high vehicular emissions in this city. Approximately 25% of the 2.2 million residents of this city live in close proximity to major arterial roadways and traffic emissions account for almost 46% of the annual PM emissions [33]. A large number of vehicles operating continuously in the city (410,000 as per 2011 estimates), 1302 industrial point sources, high sulfur content in the fuel (500 ppm for diesel and 2000 ppm for gasoline), are believed to be the major contributors for the deteriorating air quality in this mountainous city [34]. 

Previous studies in this city have examined the spatiotemporal variations of polycyclic aromatic hydrocarbons [35], and health associations between carbon monoxide and elevated carboxyhemoglobin (COHb) levels in school children [36]. More recently our research group has shown that long term exposure to traffic emissions for residents living in close proximity to major roadways could lead to increased carotid intima-media thickness (cIMT) in a cohort of school going children as measured by detectable arterial remodeling [29]. In addition, we reported the characterization of various PM species in three different neighborhoods impacted by varying levels of traffic density [37]. However, elemental composition of PM_2.5_ has not been studied or reported before in this city. We address this major research gap in Quito air quality by characterizing the elemental composition of PM_2.5_ at various schools and residences in these three low income neighborhoods of Quito. 

The main aims of this study are to: (i) characterize the elemental composition of PM_2.5_ concentrations at schools and residences in three neighborhoods of Quito, Ecuador, which were impacted by varying levels of traffic density; (ii) assess the spatial contrast of the element concentrations in both indoor and outdoor microenvironments; (iii) distinguish various geogenic and anthropogenic elements by enrichment factors; and (iv) investigate the contribution of multiple sources to the element concentrations via Principal Component Analysis.

## 2. Materials and Methods

### 2.1. Study Sites and Meteorology

**Figure 1:**
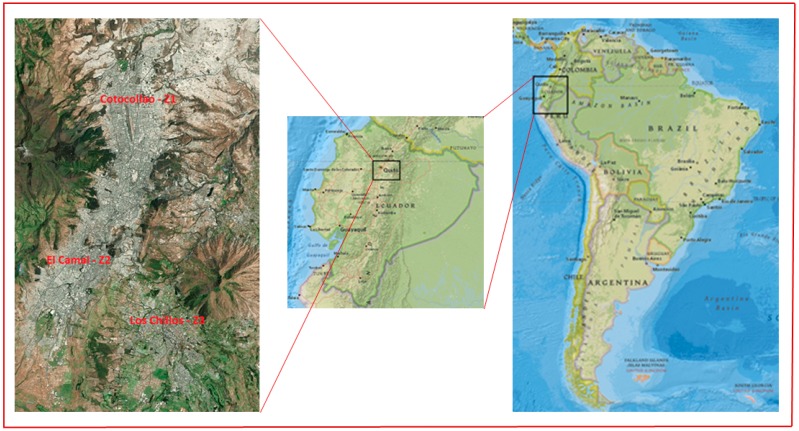
Location of the Study Sites.

Schools and residences in three economically depressed ‘low income neighborhoods’ located in the north, central, and south of the Quito Metropolitan District (QMD) were selected for the study. The selection of the low-income neighborhoods was based on historical information for the study area and in consultation with the local governmental and municipal officials. These three neighborhoods were classified as high, medium, and low exposure zones based on historical PM_10_ records collected from the three neighborhood central ambient monitoring sites [38,39]. Additionally, data on neighborhood traffic density, traffic patterns, and population characteristics were also taken into consideration. Figure 1 displays the locations of the three selected zones. The Cotocollao neighborhood in the northern part of QMD was identified as the medium exposure zone (zone 1). El Camal, located in the south central part of QMD, was designated as a high exposure zone (zone 2) whereas the southeastern neighborhood of Los Chillos was selected as low exposure zone (zone 3).

Quito is a high altitude Latin American city in the Andean mountains. The city has a subtropical highland climate with abundant sunshine year around (2000 h of sunlight per year). Temperature variation is minimal throughout the year due to its proximity to the Equator. The city experienced low northeasterly winds with speeds ranging from 1 to 2.5 m/s. Strong solar radiation due to high elevation and altitude-enhanced rapid cooling at night results in temperature inversions. The average nighttime temperatures varied from 9.3 °C to 18.7 °C. June through September is the dry season and October through May is the wet season.

One public elementary school with a minimum of 150 students located within an 8-km radius of neighborhood Central Ambient Monitoring (CAM) site was selected in each zone. The three schools, with two or three story buildings, shared common features such as a principal outdoor play area consisting of an inner cement courtyard surrounded by classrooms, each of which had direct access to an outdoor hallway. All rooms in the three schools were naturally ventilated. A random subsample of subject homes was selected for participation in each neighborhood exposure zone. They were selected based on location (within an 8-km radius of the neighborhood CAM site) and the absence of smokers in the household. All households relied on natural ventilation and opened their windows on average 7 ± 4 h per day. Between 41 and 47 homes were sampled in each of the three zones. A typical house was constructed of cement block, concrete, or steel/iron, and consisted of a kitchen/dining room, living room, two bedrooms, and a bathroom. Bottled liquefied petroleum gas (LPG) was the only cooking fuel used by households. The QMD neighborhoods in which participating subject homes were located typically included a mix of residential housing and commercial businesses, i.e., small grocery stores (“micromercados”), restaurants, bakeries, street food vendors, gasoline stations, LPG depositories, and furniture/wood working and other small shops. 

### 2.2. Sampling Plan

Seven-day indoor and outdoor PM sampling was conducted at the school and various residential sites once per month at each of the three zones for 12 months in 2010. In each zone, the school site and four (indoor and outdoor) residential sites served as controlled sites throughout the year-long monitoring period. Other residential sites varied every month between each zone, and the sampling in some homes was repeated once or twice during the study period. School indoor and outdoor PM sampling was paired. However, due to some unanticipated physical and logistical constraints, it was not always feasible to pair residential indoor and outdoor samplers. Sampling was performed subsequently at one zone per week. Indoor school sampling was done in classrooms, student’s conference room, computer room, or school director’s office. The indoor residential sampling was always performed in the living room, and within 15 m of subject homes for outdoor sampling. Indoor samplers were placed at a height of 1.2–1.8 m above ground. Outdoor samplers, both at schools and residences, were placed at a height at least 1.8 m above the ground (or on rooftops). 

### 2.3. PM_2.5_ Gravitational and Elemental Analysis

PM_2.5_ mass was collected on 37 mm diameter, 2 µm pore size polytetrafluoroethylene (PTFE) filters (Pall Life Sciences, Ann Arbor, MI, USA) using Harvard 5 LPM cascade impactors. These were custom-designed and manufactured by the Environmental Chemistry Laboratory at the Harvard University School of Public Health (Cambridge, MA, USA). The cascade impactor consists of two impaction stages (PM_10+_, PM_10−2.5_). These impaction stages are equipped with slit-shaped acceleration nozzles. PM_10+_ and PM_10−2.5_ were collected on individual polyurethane foam (PUF) impaction plugs. MEDO Pumps (Model No. VP0125, Medo USA, Inc., Roselle, IL, USA) were used to generate a constant air stream of 5 L/min into the cascade samplers. Gravimetric analysis of PM samples was conducted at the University of Texas at El Paso (UTEP) Air Quality Laboratory. Filters and PUFs were conditioned, pre-weighed and stored in Petri dishes for a period no greater than 30 days, prior to being placed into the PM samplers. The deployed filter media from each week’s sampling period were collected, identified, and stored in Ziploc^®^ bags(S. C. Johnson & Son, Inc., Racine, WI, USA) and transported to the Biomedical Research Center laboratory at the Central University of Ecuador medical school for storage until transport to UTEP for post-weighing. The gravimetric analyses used in the current study are described in detail elsewhere [40]. 

After the completion of the gravitational analyses, the PM_2.5_ filters were analyzed for elemental composition with an Inductively Coupled Plasma Mass Spectrometry (ICP-MS). The instrumentation used for this analysis was the ICP-MS Hewlett Packard 4500 (Agilent Technologies, Inc., Santa Clara, CA, USA). Complete details about the methodology are described in detail elsewhere [41]. A total of 48 elements were determined through this method; however, only 40 of these were included in the analysis because the remaining 8 elements were below the detection limits. These 40 elements are as follows: Na, Mg, Al, Si, S, K, Ca, Sc, Ti, V, Cr, Mn, Fe, Co, Ni, Cu, Zn, As, Se, Rb, Sr, Y, Mo, Rh, Pd, Ag, Cd, Sn, Sb, Te, Cs, Ba, W, Pt, Au, Pb, Bi, La, Th. The detection limits of these elements are shown in Table 1. Certified Reference Material (CRM) from the National Institute of Standards & Technology (NIST, Gaithersburg, MD, USA) were used for calibration purposes. The recovery rates of all the elements ranged from 80% to 120% with variation below 10%. The detection limit was calculated from 10 replicate measurements of a Teflon blank sample and based on one standard deviation.

### 2.4. Statistical Data Analysis

Descriptive statistics of the data were generated using IBM SPSS (version 22.0, IBM Corp., Armonk, NY, USA) and Microsoft Excel 2007 (Microsoft Inc., Redmond, WA, USA). Statistical significance was defined as *p* < 0.05. Box-plots were used to characterize elemental concentrations across the various school and residential sites in both the indoor and outdoor microenvironment. The boxes are the inter-quartile ranges (75th & 25th), the whiskers show the minimum and maximum values, the outliers designated with circles are values between 1.5 and 3 box lengths from either end of the box, and extreme values, designated as asterisks, are values more than 3 box lengths from either end of the box. The median is indicated by the thick black line inside the boxes. Inter-element correlations were computed at each of the three sites using Spearman’s Rho correlations. Enrichment Factors, and Principal Component Analysis helped determine the origination of various sources that contributed to the elemental concentration loadings. 

## 3. Results

### School and Residences Elemental Concentrations

**Figure 2:**
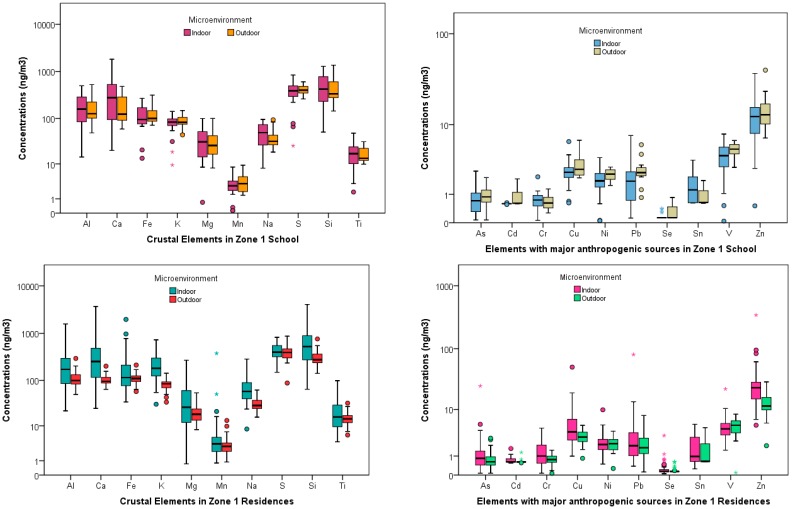
Elemental concentrations at school and residences in Zone 1.

**Figure 3:**
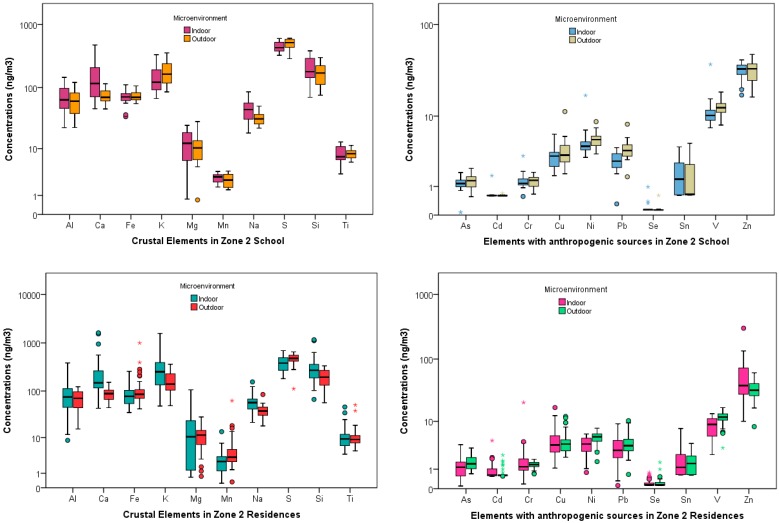
Elemental concentrations at school and residences in Zone 2.

**Figure 4:**
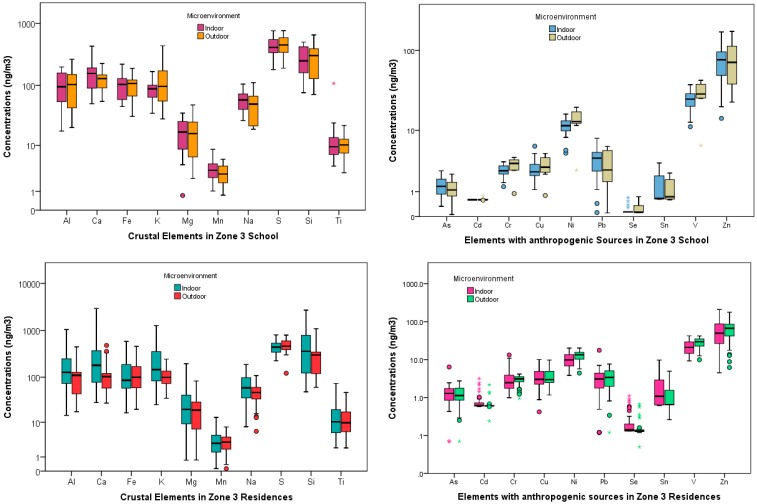
Elemental concentrations at school and residences in Zone 3.

Table 2 and Table 3 show the basic statistics for the element concentrations at the schools and residences (both indoors and outdoors) for the three zones, respectively. Al, Ca, Fe, K, Mg, Mn, Na, S, Si, T are typically considered to be of natural origin such as earth crustal, volcanic or soil tracers. As, Cd, Cr, Cu, Ni, Pb, Se, Sn, V, Zn are elements that are usually attributed to anthropogenic sources [42]. These elements are primarily emitted from vehicles (tailpipe, brake and tire wear, engine abrasion), industrial facilities (heavy metal industries, smelting operations, fuel and coal combustions), and uncontrolled waste incineration and biomass burning. Spatial contrast between these two subgroup of elements for the schools and residences in both the indoor and outdoor microenvironment at the three zones are shown in the boxplots (Figure 2, Figure 3 and Figure 4). 

Mass concentrations for PM_2.5_ across the schools and residences in the three zones have been discussed extensively by the authors elsewhere [37] Amongst the geogenic elements, Silica (Si) was the most abundant at zone 1 school for the indoor, 524.08 (360.25) ng/m^3^, and outdoor, 535.58 (432.47) ng/m^3^, microenvironment. At the residential homes in this zone, the indoor concentration, 726.24 (759.68) ng/m^3^, was greater than the outdoor concentrations, 308.28 (115.27) ng/m^3^. These high concentrations were expected as there are many quarries a few miles north of zone 1 sampling area. The concentration of this element at the zones 2 and 3 schools were: {Indoor: 204.19 (93.08) ng/m^3^, Outdoor: 173.23 (78.16) ng/m^3^} and {Indoor: 276.60 (141.20) ng/m^3^, Outdoor: 290.83 (179.77) ng/m^3^}, respectively. Similar pattern for Si concentrations was also observed at the residences in these two zones with both the indoor and outdoor concentrations being higher in zone 3 {Indoor: 508.89 (530.59) ng/m^3^, Outdoor: 312.47 (233.59) ng/m^3^} compared to zone 2 {Indoor: 346.62 (275.05) ng/m^3^, Outdoor: 198.13 (77.35) ng/m^3^}. Sulfur (S) was found to be the most abundant element at the schools and residences in both zones 2 and 3. At zone 2, the school outdoor concentration, 503.43 (101.78) ng/m^3^ exceeded the indoor concentration, 447.63 (87.53) ng/m^3^. The indoor concentration, 437.22 (144.75) ng/m^3^ was less than the outdoor concentration, 463.19 (162.04) ng/m^3^, in zone 3 school. It is plausible that the loadings for this element were influenced by some anthropogenic sources such as diesel emissions or biomass combustion in addition to natural sources such as secondary sulfates in these two zones. 

The next abundant element at the three zones was Ca, followed by Al. The concentrations for these two elements also mirrored the pattern for Si across the three zones with school concentrations being the highest at zone 1 {Ca: 406.64 (424.17) ng/m^3^ (Indoors), 193.97 (146.32) ng/m^3^ (Outdoors); Al: 192.49 (135.38) ng/m^3^ (Indoors), 198.51 (165.78) ng/m^3^ (Outdoors)} followed by zone 3 {Ca: 164.12 (108.28) ng/m^3^ (Indoors), 124.99 (49.40) ng/m^3^ (Outdoors); Al: 102.38 (59.31) ng/m^3^ (Indoors), 106.01 (74.12) ng/m^3^ (Outdoors)} and zone 2 {Ca: 163.27 (134.24) ng/m^3^ (Indoors), 75.27 (23.10) ng/m^3^ (Outdoors); Al: 71.75 (36.38) ng/m^3^ (Indoors), 63.66 (33.68) ng/m^3^ (Outdoors)}. The concentrations for Al, Ca, and Si suggest that the geogenic sources such as crustal and soil tracers are predominant in zone 1 (Cotocollao) and zone 3 (Los Chillos) in contrast to zone 2 (El Camal). This was expected because zone 1 is impacted by quarries as mentioned above and zone 3 is located near a dormant volcano. 

The concentrations of the other geogenic elements in zone 1 school were ranked in the following order: Fe > K > Na > Mg > Ti > Mn. However, at the residences in this zone, K exceeded Fe and other elements following the same rank order as zone 1 school. In zone 2 school, the indoor, 152.00 (76.25) ng/m^3^, and outdoor, 186.54 (90.51) ng/m^3^ potassium (K) concentrations exceeded that of iron (Fe) indoors and outdoors, 70.12 (19.19) ng/m^3^, 76.10 (17.23) ng/m^3^, respectively. However, in zone 3 school, the concentrations of K, 129.81 (118.55) ng/m^3^ exceeded that of Fe, 100.53 (43.80) ng/m^3^, in the outdoor microenvironment but not indoors {Fe, 99.73 (47.56) ng/m^3^ > K, 90.79 (39.23) ng/m^3^}. Similar patterns for these two elements were observed at the zones 2 and 3 residential homes.

Amongst the anthropogenic subgroup elements, the concentrations of Zn were the highest followed by V across the three zones for the schools and residences both indoors and outdoors. At the zone 3 school, Zn concentrations were the highest {Indoor: 77.17 (42.76) ng/m^3^, Outdoor: 77.12 (48.50) ng/m^3^} followed by zone 2 {Indoor: 31.99 (7.35) ng/m^3^, Outdoor: 32.48 (9.96) ng/m^3^} and zone 1 {Indoor: 13.19 (7.92) ng/m^3^, Outdoor: 15.60 (9.77) ng/m^3^}. V and Ni concentrations followed the same trend (zone 3 > zone 2 > zone 1) for both indoors and outdoors at schools and residences. Vanadium (V) concentrations at the schools ranged from 30.14 (10.50) ng/m^3^ (zone 3, outdoors) to 4.04 (1.98) ng/m^3^ (zone 1, indoors). The residential concentrations for V followed the same patterns as well with the highest value at zone 3 outdoors, 28.43 (8.23) ng/m^3^, and the least concentrations at zone 1 indoors, 4.88 (3.25) ng/m^3^. Also, in this subgroup Se recorded the lowest concentration for both the residences and school indoor and outdoor locations.

## 4. Discussion

### 4.1. Indoor-Outdoor Relationships at the Three Schools

**Figure 5:**
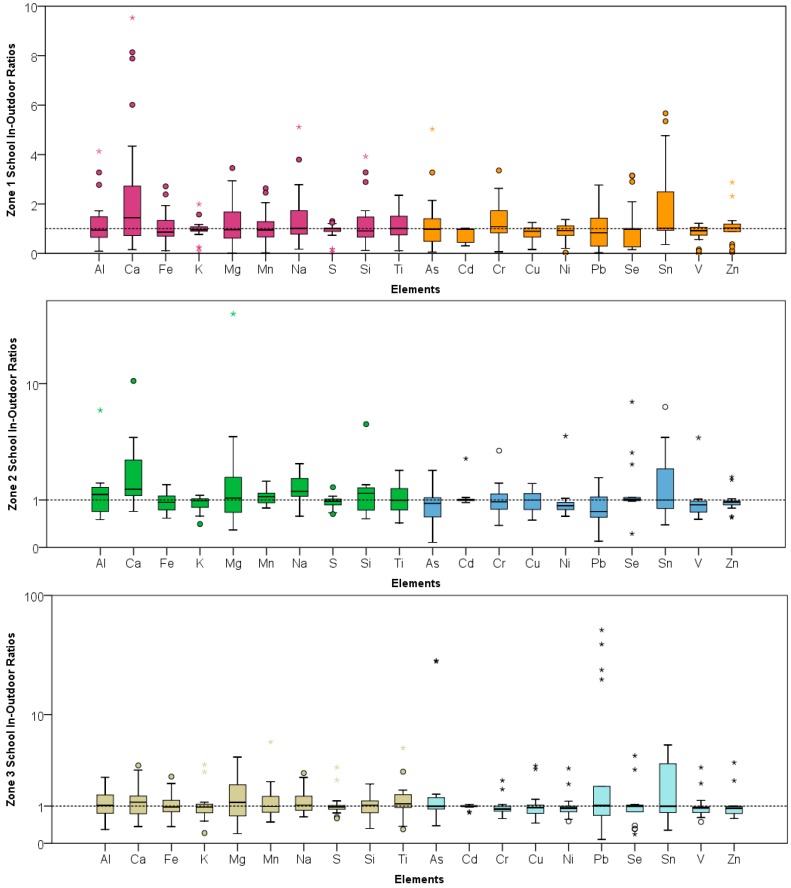
Indoor-Outdoor Ratio boxplots at the three schools.

Indoor-outdoor (I/O) ratios were computed for the elements analyzed in this study to understand the role of various pollutant sources in both these microenvironments. The mean I/O ratios along with the various statistical parameters are shown in Table 4 for the schools in the three zones. Spatial contrast for the I/O ratios of twenty elements in the three zones is also plotted and shown in Figure 5. I/O ratios were only computed for the schools as the measurements in the indoor and outdoor microenvironment were paired and conducted concurrently. Only one central outdoor sample was collected for each zone; therefore, I/O ratios were not reported for the residences. Also, samples were collected in more than one indoor locations, especially in zone 1 school, during some sampling sessions. Therefore, the number of paired in-outdoor samples is greater than 10 for the zone 1 school. Indoor-outdoor Paired Sample t-test was also computed to assess the statistical significance of the concentration differences between these two microenvironments. 

In zone 1, I/O ratios for predominantly anthropogenic elements ranged from 0.78 (0.97) for Cd to 2.04 (1.02) for Sn (*p* value < 0.05). In zone 2, the I/O ratios for Ca, K, and Na were 2.31 (2.53), 0.89 (0.21), and 1.39 (1.27), respectively and were also statistically significant (*p* < 0.05). In zone 3, the ratios for the 10 crustal elements were greater than 1 suggesting the role of various indoor sources for these elements in the school. Schools in the three zones were all naturally ventilated. Heavy foot traffic in the classrooms might have contributed to these high ratios. I/O ratios were the highest for Mg in zone 2 {3.26 (7.65)} and Ca in zone 1 {2.61 (2.86)}. I/O ratios for As {4.23 (9.23)} and Pb {8.65 (15.84)} in zone 3 suggest the possible role of some indoor sources. Indoor lead has been attributed to burning of incense sticks [43] and candles. Lead is used as a stiffening agent in the core of candle wicks to help it stay out of the molten wax resulting in its emission and deposition on indoor furniture [44].

### 4.2. Inter-Element Correlation Relationships

Spearman’s correlation coefficients were computed to assess the various inter-element relationships across the three zones. Correlations 0.6 and above were considered to be strongly associated with each other and are shown in Table 5. The *p*-value was set at 0.05 level. Correlation coefficients help understand the temporal relationships between the various studied elements. Across the three zones, Ni was very strongly correlated with V (0.93 < *r* < 0.99) confirming a common source (heavy industrial oil and pet coke combustion) for these two elements [45,46,47]. Strong relationships were also observed between the various crustal elements. In zone 1, Al, Ca, Fe, Mg, Na, Si, and Ti were all very robustly correlated with one another (0.65 ≤ *r* ≤ 0.99). Sulfur was very weakly correlated (*r* < 0.25) with the above elements suggesting that it is associated with sulfate of secondary origin such as pet coke combustion, diesel powered vehicles, and some other non-geological sources [48].

In zone 2, Si was very strongly correlated with Al (0.91), Ca (0.87) followed by Na (0.70), Mg (0.64), Fe (0.63), and Ti (0.70). These robust relationships demonstrate the dominating role of natural sources such as soil tracers and crustal earth metals. Similar to zone 1, zone 3 crustal elements Al, Ca, Fe, Mg, Mn, Na, Si, Ti exhibited very strong and statistical relationships with each other (0.6 < *r* < 0.96, *p* < 0.001). Sulfur was sparsely or scarcely correlated with these elements—a pattern observed in zone 1 as well. The pairings of S with Cr, Ni, and V in zone 3 yielded a correlation value of *r* = 0.61. Cr and S in zone 3 could be attributed to fuel combustion by industries and motor vehicles. Similarly, in zone 2 the r value of 0.69 (S-Ni), and 0.73 (S-V) suggest common sources. Cr and Ni—tracer of pyro-metallurgical processes such as non-ferrous metal industries and steel plants were also related to each other (*r* = 0.68) in zone 1 [49,50,51].

As and Pb were robustly related to each other {zone 2 (0.74); zone 3 (0.86)}. Also, Pb and Zn in zone 3 exhibited a strong relationship (*r* = 0.73) suggesting the role of vehicular emissions [52] in addition to waste incineration emissions [53]. Cu and Mn exhibited a strong relationship (*r* = 0.62) suggesting possible role of traffic emissions in zone 1 [54]. Relationship between these two elements was also observed in zone 3 (*r* = 0.68). There are multiple sources of copper in the urban environment. Emissions from brake linings and tire wear [55], and various industrial processes [56] are some of the pathways that Cu can enter the environment. In brief, the Spearman’s correlation relationships observed in this study across the three zones implies that there are multiple sources in Quito for the PM and the necessity for building source profiles to develop effective mitigation measures. In addition to the inter-element correlation analyses, we have attempted to identify the major sources for the PM pollution in the city through the enrichment factor analysis and principal component analysis (PCA) as discussed below.

### 4.3. Enrichment Factor Analysis

**Figure 6:**
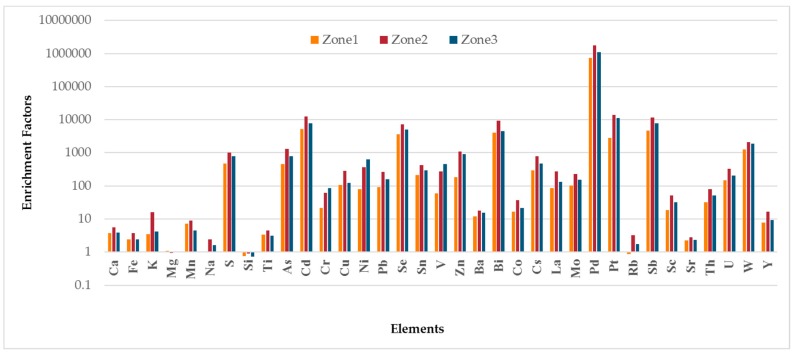
Enrichment Factors for the studied elements in the three zones.

The strength of the crustal and non-crustal sources was evaluated by calculating the Enrichment Factor (EF) for the elements quantified in this study. EF provides an estimation of the amount of an element that is in excess compared to the concentration of the reference element. Aluminum was chosen as the reference element for calculating these EFs using upper continental crust data [57]. EF is calculated by using the following formula:

EF_i_ = {c_i_/c_Al_]_air_/[c_i_/c_Al_}_crust_

where c_i_ is the mass concentration of element i and c_Al_ is the concentration of the reference element aluminum. The subscripts ‘air’ and ‘crust’ indicate that the ratios were obtained from the concentrations of element i and reference element Al in the air and upper continental crust, respectively. EF < 10 suggests the enrichment of elements by natural geological sources. EF between 10 and 100 indicates enrichment by anthropogenic sources in addition to crustal sources and EF > 100 indicates anthropogenic sources are the major enrichment contributors for these elements [58]. One notices that the thresholds used by these authors are optimal and other thresholds, especially for moderate enrichments (both anthropogenic and crustal), have been used by other researchers [59]. The EF values were displaced in a logarithmic scale for the elements at the three zones as shown in Figure 6.

Ca, Fe, Mg, Mn, Na, Si, Ti had EF less than 10 in each of the three zones suggesting that the dominant source for these elements is crustal in origin. In zone 2, the EF for K was around 16 demonstrating the influence of non-crustal sources toward its enrichment. The EF for this element in zones 1 and 3 was less than 5. S had high EF values (zone 1: 476, zone 2: 1032, zone 3: 776) suggesting that the majority of the source loadings for this element in the three zones was anthropogenic such as pet coke combustion and diesel emissions. EF values in zone 1 for As, Cd, Cr, Cu, Ni, Pb, Se, Sn, V, and Zn were always less than zones 2 and 3. Emission patterns for these elements in the ambient environment confirm the role of industrial and vehicular emissions toward their loadings. EF for As (1296) and Pb (266) were the highest in zone 2 in contrast to the other two zones. EF values for Ni and V in zone 3 (628, 458) were higher than zone 2 (368, 273), respectively. The possible explanation for this observation is the role played by the two thermoelectric plant in zone 3 that uses high amounts of pet coke and diesel. As, Cd, Cu, Ni, Pb, Se, Sn, Sn, V, and Zn were all significantly enriched (EF > 100) demonstrating the role of non-crustal sources for their presence in ambient air in both zones 2 and 3. 

EF for Cr suggest moderate enrichment (zone 1: 22, zone 2: 62, zone 3: 85). This hints toward oil combustion mixed with soil dust [53]. Across the three zones, the EF for Cd was greater than 5000 confirming the strong influence played by industrial activities [60] and waste incineration [61] toward its enrichment. EF for Rb (0.89, 3.25, 1.77) and Sr (2.24, 2.83, 2.33) in zones 1–3, respectively, confirms that the origin of these two elements in the ambient environment is due to crustal/geological sources [62]. Co is emitted through oil combustion and vehicular sources [63] and Ba could be attributed to traffic related emissions [55]. This was obvious by their moderate EF values—Co {16.42 (Z1), 37 (Z2), 21 (Z3)} and Ba {12 (Z1), 18 (Z2), 16 (Z3)}. Ba can originate from multiple sources in an urban environment. It is used in lubricants to minimize engine abrasion in diesel powered vehicles [64] and is considered a source of road dust [65]. Road dust comprises of particles from unpaved roads, motor transportation generated resuspended dust, and dust emanated from the abrasion of tires and brake linings [66]. 

Although Co and La are regarded as rare earth metals and are typically associated with geological dust, many studies have suggested that these two elements can be a marker for environmental tobacco smoke (ETS), especially in indoor and outdoor micro-environments [64,67,68,69]. As per our records, none of the study participants houses or schools had any smokers. However, it stands to reason that the natural ventilation in schools and residences and crowded living conditions could have resulted in the dispersion of ETS in the various microenvironment. The EF for La {Zone 1: 87, Zone 2: 271, Zone 3: 131} suggests the existence of possible fugitive emission sources in zones 2 and 3. 

The EF for Sb were the highest in zone 2 (11,438) followed by zone 3 (7889) and zone 1 (4648). Sb emissions in an urban environment can be attributed to a suite of factors: vehicular emissions [70], waste incineration [53], oil combustion and vehicle sources [63], brake dust [63,71,72], and emissions from brake lining, brake pads, and tire wear [55,73]. The high EF for this element, therefore, suggests that industrial and vehicular emissions are the strongest sources for its emission in the environment. EF for Bi were >4000 in zones 1 and 3, and 9323 at zone 2. Bismuth finds many applications in the industry—chiefly medicines, cosmetics (lipstick), paint and pigments, semiconductors, and as an alloy in many metallurgical operations [74]. An industrial park (5.9 miles south of El Camal-zone 2) has myriad types of facilities such as smelters, paints and adhesives manufacturing, and paint factories in Cotocollao (zone 1) could be some of the emission sources for Bi in the Quito ambient air. 

The EF for two of the platinum group elements (PGE), Pd (Palladium), and Pt (Platinum) warrants further discussion here. The EF for Pd was the highest amongst the elements analyzed in this study: zone 1 (7.3 × 10^5^), zone 2 (1.7 × 10^6^), zone 3 (1.1 × 10^6^) and EF for Pt at the three zones were: Z1: 2793, Z2: 14,078, Z3: 11,206. The usage of these two elements has increased considerable in the last few years due to its important application as exhaust catalysts in automotive catalytic convertors [75,76]. These convertors have been very instrumental in reducing the burden of CO, NO_x_ and other pollutants in the environments; however, the concentration of these PGE in air is increasing considerably [77]. In addition, these elements also find usage in other industrial applications such as the pharmaceutical and chemical industry. The enrichment of these two elements in the QMD could, therefore, be strongly attributed to their usage in catalytic convertors and other such industries. 

Findings from this study suggests that elements with high concentrations such as Ca, Si have low enrichment factors (<10) and elements such as Pt, Pd with very high EFs (>10 × 10^3^) usually have very low concentrations. This suggests that the overall contribution of elements such as Pt and Pd is anthropogenic and the contribution of natural sources toward their enrichment is non-existent.

### 4.4. Principal Component Analysis

Source apportionment of twenty elements in the PM_2.5_ was carried out by using Principal Component Analysis (PCA) with varimax rotation. Four to six factors with Eigen values greater than unity were obtained for each of the three zones. Factor loadings equal to or greater than 0.5 are shown in bold typeface and correspond to statistical significant variables and are presented in Table 6. Valid samples from both the indoor and outdoor microenvironment at the schools and residences across the three zones were included in the PCA analysis. 

Five principal components accounted for 75% of the total variance in zone 1. The first factor {F1, 34.6% of the variance} was characterized by Al (0.98), Ca (0.90), Mg (0.97), Na (0.65), Si (0.98), and Ti (0.82). The high loadings for these elements show the strong dominance of crustal sources. The second factor {F2, 13.9% of the variance} had very high loads for Fe (0.87), Mn (0.98), and Cu (0.85), which could possibly be attributed to a secondary crustal contribution. The variance of the third, fourth, fifth, and sixth factors were 11.51%, 8.85%, 5.62%, and 5.12%, respectively. These high loadings could be attributed to a combination of both natural and anthropogenic sources such as local and regional dust resuspended by wind transportation, and convection processes [54,78], and traffic emissions [55]. Factors 3 and 4 in this zone were represented by high loadings of Ni (0.93)-V (0.97) and As (0.97)-Pb (0.98), respectively. As explained before, Ni and V are signatures of heavy fuel and oil and pet coke combustion and. Pb was banned from gasoline in 1998; however, the high loadings suggest that soil acts as a reservoir for historic lead and could be emitted into the various environmental media during the resuspension of soil and road dust. Both Pb and As are typical signatures for non-ferrous metallurgical operations [79,80] and municipal waste incineration [81]. Cr (0.57) and Zn (0.78) represented the fifth factor, possibly characteristics of activities associated with industrial combustions. There are multiple sources of Cr and Zn in the environment. Cr could enter the environment by, sewage sludge incineration [82] and pyro-metallurgical processes [49]. Zn could be attributed to motor vehicle traffic, non-ferrous smelters, tire wear and tailpipe emissions due to its usage in engine oil [83] in addition to industrial metallurgical processes [84]. 

In zone 2, six factors were retrieved from the PCA analysis explaining 78% of the variance. Similar to zone 1, the crustal elements Al (0.97), Ca (0.90), Mg (0.89), Na (0.54), Si (0.95), Ti (0.57) comprised of the first factor with the largest variance (27.76%). Factor 2 explained 14.36% of the variance and was represented by K (0.88) and Na (0.69). The F2 loading for Na was a little higher than F1 (0.54) suggesting the possible influence of marine aerosols [85] than geological crustal sources. Marine aerosols could play a role in high altitude Quito region, perhaps, due to the thermal differences in the oceanic water and various atmospheric layers resulting in the long-range transport of these particles across the marine-atmospheric boundary layer interface [86]. Ni (0.91), V (0.92), S (0.79) formed the third factor explaining 9.47% of the variance. S is usually associated with biomass burning, diesel emissions, pet coke combustion and the grouping of these three elements hints at predominant combustion processes that comprises of both vehicular (gasoline and diesel) and heavy oil emissions. The El Camal region of QMD is historically considered as one of the most polluted zones with a heavy traffic density and concentration of many industrial establishments. F5 {Fe (0.96), Mn (0.98)}, F6 {Cr (0.76), Sn (0.86)}, and F7 {Ti (0.57), Se (0.95)} explained 7.73%, 6.25%, and 5.33% of the variance, respectively. The high loadings for Fe and Mn in F5 suggest the role played by both traffic and metallurgical processes [84]. The high loadings of Sn and Cr in Factor 6 represent industrial emissions. Sn in an urban environment is emitted usually through smelter operations and metal production facilities [87] and Cr in this zone could be attributed to a pigment and paint manufacturing plant [81].

Four factors explained 73% of the total variance in Zone 3. Factor F1 (43% of the variance) accounted for nine elements: Al (0.94), Ca (0.79), Fe (0.86), Mg (0.93), Mn (0.71), Na (0.64), Si (0.93), Ti (0.76), Cu (0.55). High loadings for Al, Mg, Ti (>0.90) explain the role of crustal sources similar to the other two zones. The moderate loading of Cu (0.55) in this factor suggest the possible role of resuspended soil dust along with natural soil markers. The second factor {F2, 12.07% of the variance} represented moderate loadings of Mn (0.52), Na (0.62) and high loadings of As (0.84), Pb (0.91), Zn (0.82). This factor was influenced by multiple sources, both natural and anthropogenic. Similar to zone 2, F3 accounted for S (0.73), Ni (0.95), V (0.96). Cd (0.60), K (0.86), Cr (0.69) were represented by Factor 4 explaining 7.42% of the variance. K and Cr could be associated with wood burning [88] and Cd can be emitted from multiple sources in the environment such as vehicle emissions [60], waste incineration [53], and metallurgical processes [54]. 

## 5. Conclusions

We present findings from the elemental characterization of PM_2.5_ at schools and residences impacted by different traffic densities at three low income neighborhoods in Quito, Ecuador. To the best of our knowledge, this is the first study in the Ecuadorian capital that has explored the elemental speciation of fine particulate matter thereby bridging this very important research gap in the Quito air quality literature. Across the three zones, the various elements exhibited very heterogeneous patterns in their concentrations implying the role of multiple sources of fine particulate air pollution. As expected, Si was the most abundant geogenic element with the highest concentrations recorded at medium traffic density neighborhoods at zone 1 (Cotocollao) area, followed by low traffic density zone 3 (Los Chillos) and high traffic density zone 2 (El Camal) neighborhoods. Zones 1 and 3 were impacted by re-suspension of dust from unpaved surfaces and roads, soil erosion, and fugitive emissions from quarries as corroborated by the EF and PC analysis. Amongst the anthropogenic sub-group of elements, concentrations of zinc were the highest followed by V, Ni, and Pb. Concentrations of these elements differed considerably across the different zones with the Los Chillos—zone 3 region exhibiting the highest concentrations at both the schools and residences as well as both the microenvironments. In-outdoor ratios suggested the role of both indoor sources and infiltration of elements from outdoors into the indoor microenvironment. This was expected due to natural ventilation at all the schools and residences. 

Inter-elemental correlations were computed for the studied elements at the three zones. Geogenic elements such as Al, Si, Ca, K, Na, were all very high correlated with each other implying the dominating role of soil erosion, and crustal sources toward their concentrations. S was weakly correlated with these elements indicating that in addition to natural geological sources, biomass and pet coke combustion also contributed toward its concentrations. Ni and V were very strongly correlated across the three zones confirming the role of heavy fuel and oil and pet coke combustion in the Quito metropolitan region. As and Pb were also correlated with other each and also with Zn suggesting the role of multiple sources such as vehicular and industrial emissions. Albeit, zone 2 region was very heavily impacted by traffic and industrial emissions, the concentrations of many anthropogenic sourced elements such as Pb, As, Cr, Ni, V, and Zn were higher in zone 3. This could be attributed to the two thermal power plants that used low grade heavy oil and pet coke for electricity generation in zone 3. 

Enrichment Factors were also calculated for the studied elements. Geogenic elements, except S, had values <10, and the anthropogenic subgroup elements had EF >10. High enrichment factors and low concentrations, especially for Zn, V, Ni, As, Pb implied the role played by industrial and vehicular emissions toward their contribution in the Quito urban air shed. Multivariate statistical analyses such as Principal Component Analysis was undertaken to investigate the source apportionment of these studied elements. Resuspended road dust, diesel and gasoline emissions, brake and tire wear, pet coke and oil combustion, metallurgical and other industrial emissions were identified as some of the major sources for the elemental concentrations. The findings from the PCA also complimented the observations from Enrichment Factor analysis and Inter-element correlations. It is our firm belief that these study results would aid the Ecuadorian policy makers in addressing the pressing topic of rising fine particulate air pollution in the capital city and help formulate policies that would minimize the exposure of sensitive populations like young children, especially in the high mountainous belt of western part of the Latin American continent. 

## Figures and Tables

**Table 1:** Detection Limits (DL) of the studied elements through the ICP-MS analysis (ng/m^3^).

Elements	DL
Na	0.0085
Mg	0.0106
Al	0.0089
Si	0.0044
S	0.0010
K	0.0011
Ca	0.0017
Sc	0.0034
Ti	0.0010
V	0.0006
Cr	0.0009
Mn	0.0020
Fe	0.0020
Co	0.0005
Ni	0.0008
Cu	0.0013
Zn	0.0014
As	0.0010
Se	0.0020
Rb	0.0007
Sr	0.0013
Y	0.0011
Mo	0.0028
Rh	0.0033
Pd	0.0065
Ag	0.0069
Cd	0.0088
Sn	0.0094
Sb	0.0086
Te	0.0102
Cs	0.0117
Ba	0.0127
W	0.0222
Pt	0.0056
Au	0.0046
Pb	0.0018
Bi	0.0043
La	0.0189
Th	0.0053

**Table 2:** Element concentrations at the schools in the three zones.

Zones	Z1		Z2		Z3	
Schools	Indoor (*n* = 23)	Outdoor (*n* = 10)	Indoor (*n* = 16)	Outdoor (*n* = 11)	Indoor (*n* = 19)	Outdoor (*n* = 10)
Elements	Mean ± SD	Mean ± SD	Mean ± SD	Mean ± SD	Mean ± SD	Mean ± SD
PM_2.5_	10.70 ± 4.94	10.87 ± 3.23	14.66 ± 15.64	13.19 ± 3.47	10.83 ± 8.85	12.98 ± 8.71
Al	192.49 ± 135.38	198.51 ± 165.78	71.75 ± 36.38	63.66 ± 33.68	102.38 ± 59.31	106.01 ± 74.12
Ca	406.64 ± 424.17	193.97 ± 146.32	163.27 ± 134.24	75.27 ± 23.10	164.12 ± 108.28	124.99 ± 49.40
Fe	123.65 ± 68.58	139.14 ± 87.90	70.12 ± 19.19	76.10 ± 17.23	99.73 ± 47.56	100.53 ± 43.80
K	80.85 ± 31.05	88.58 ± 31.25	152.00 ± 76.25	186.54 ± 90.51	90.79 ± 39.23	129.81 ± 118.55
Mg	37.00 ± 28.35	36.67 ± 30.76	12.49 ± 7.56	11.16 ± 7.68	17.87 ± 11.17	18.25 ± 15.21
Mn	3.15 ± 2.07	3.86 ± 2.46	2.81 ± 0.66	2.51 ± 0.91	3.55 ± 1.72	2.98 ± 1.51
Na	49.42 ± 28.40	41.48 ± 25.31	45.74 ± 18.08	32.93 ± 9.45	60.47 ± 23.14	53.02 ± 30.18
S	400.55 ± 208.62	412.45 ± 99.56	447.63 ± 87.53	503.43 ± 101.78	437.22 ± 144.75	463.19 ± 162.04
Si	524.08 ± 360.25	535.58 ± 432.47	204.19 ± 93.08	173.23 ± 78.16	276.60 ± 141.20	290.83 ± 179.77
Ti	18.69 ± 11.60	17.74 ± 7.99	8.18 ± 2.86	8.24 ± 1.82	15.57 ± 23.01	10.67 ± 5.13
As	0.83 ± 0.71	0.91 ± 0.55	1.09 ± 0.46	1.29 ± 0.48	1.36 ± 0.59	1.17 ± 0.64
Cd	0.59 ± 0.02	0.91 ± 0.50	0.66 ± 0.25	0.61 ± 0.03	0.60 ± 0.01	0.61 ± 0.06
Cr	0.75 ± 0.42	0.66 ± 0.31	1.32 ± 0.57	1.25 ± 0.34	2.58 ± 0.63	3.15 ± 0.96
Cu	2.49 ± 1.13	3.25 ± 1.55	3.27 ± 1.26	4.10 ± 2.65	2.84 ± 1.22	3.07 ± 1.19
Ni	1.83 ± 0.91	2.22 ± 0.51	5.15 ± 3.32	5.39 ± 1.51	11.27 ± 3.15	13.78 ± 4.96
Pb	2.12 ± 1.89	2.67 ± 1.36	2.75 ± 1.01	4.05 ± 1.69	3.82 ± 2.09	3.03 ± 2.12
Se	0.18 ± 0.10	0.31 ± 0.24	0.22 ± 0.22	0.18 ± 0.14	0.21 ± 0.16	0.25 ± 0.20
Sn	1.52 ± 1.05	0.93 ± 0.46	1.55 ± 1.08	1.70 ± 1.51	1.28 ± 0.94	1.18 ± 0.69
V	4.04 ± 1.98	4.95 ± 1.20	11.95 ± 7.13	12.73 ± 3.07	24.81 ± 6.89	30.14 ± 10.50
Zn	13.19 ± 7.92	15.60 ± 9.77	31.99 ± 7.35	32.48 ± 9.96	77.17 ± 42.76	77.21 ± 48.50
Ag	0.51 ± 0.22	0.49 ± 0.06	0.47 ± 0.01	0.47 ± 0.02	0.47 ± 0.01	0.48 ± 0.04
Au	0.50 ± 0.31	0.77 ± 0.65	0.50 ± 0.35	0.72 ± 0.66	0.84 ± 0.75	0.45 ± 0.41
Ba	7.23 ± 5.01	11.17 ± 6.78	4.91 ± 3.79	8.57 ± 5.03	7.51 ± 5.45	7.52 ± 4.82
Bi	0.50 ± 0.35	0.51 ± 0.45	0.47 ± 0.32	0.73 ± 0.63	0.40 ± 0.31	0.54 ± 0.39
Co	0.14 ± 0.21	0.30 ± 0.76	0.12 ± 0.22	0.04 ± 0.02	0.16 ± 0.36	0.05 ± 0.07
Cs	1.08 ± 1.12	1.35 ± 0.90	1.33 ± 1.08	0.97 ± 0.53	1.34 ± 1.08	0.91 ± 0.26
La	3.07 ± 2.61	2.70 ± 1.81	4.70 ± 3.51	2.75 ± 2.04	3.36 ± 2.35	3.70 ± 2.43
Mo	0.19 ± 0.00	0.20 ± 0.03	0.19 ± 0.01	0.20 ± 0.01	0.19 ± 0.00	0.22 ± 0.08
Pd	0.53 ± 0.46	0.52 ± 0.18	0.50 ± 0.21	0.65 ± 0.67	0.54 ± 0.42	0.54 ± 0.26
Pt	1.54 ± 1.08	1.52 ± 1.44	3.02 ± 1.25	3.10 ± 1.56	6.32 ± 3.50	6.58 ± 4.28
Rb	0.16 ± 0.22	0.13 ± 0.13	0.23 ± 0.24	0.21 ± 0.15	0.16 ± 0.19	0.16 ± 0.19
Rh	0.36 ± 0.28	0.26 ± 0.10	0.43 ± 0.60	0.49 ± 0.50	0.34 ± 0.34	0.36 ± 0.30
Sb	1.39 ± 1.04	0.98 ± 0.58	1.46 ± 1.30	1.24 ± 1.01	0.76 ± 0.46	0.75 ± 0.49
Sc	0.35 ± 0.29	0.35 ± 0.35	0.34 ± 0.22	0.24 ± 0.01	0.27 ± 0.14	0.28 ± 0.13
Sr	1.64 ± 1.08	1.39 ± 0.87	0.85 ± 0.58	0.74 ± 0.37	0.98 ± 0.56	0.94 ± 0.68
Te	0.99 ± 0.77	1.71 ± 1.25	1.00 ± 1.20	1.07 ± 1.19	1.50 ± 1.49	1.26 ± 0.83
Th	0.41 ± 0.18	0.51 ± 0.43	0.67 ± 0.57	0.42 ± 0.20	0.52 ± 0.47	0.48 ± 0.35
U	0.49 ± 0.49	0.37 ± 0.28	0.54 ± 0.63	0.47 ± 0.28	0.62 ± 0.70	0.44 ± 0.33
W	2.53 ± 1.87	1.57 ± 0.21	1.51 ± 0.04	2.33 ± 2.61	3.51 ± 3.34	4.62 ± 3.26
Y	0.24 ± 0.22	0.19 ± 0.18	0.25 ± 0.19	0.22 ± 0.16	0.16 ± 0.19	0.29 ± 0.24

Unit: µg/m^3^ for PM_2.5_ and ng/m^3^ for all the other elements; *n* = valid samples included in the final analysis.

**Table 3:** Element concentrations at the residences in the three zones.

Zones	Z1		Z2		Z3	
Residences	Indoor (*n* = 44)	Outdoor (*n* = 42)	Indoor (*n* = 44)	Outdoor (*n* = 43)	Indoor (*n* = 40)	Outdoor (*n* = 41)
Element	Mean ± SD	Mean ± SD	Mean ± SD	Mean ± SD	Mean ± SD	Mean ± SD
PM_2.5_	28.95 ± 30.49	12.48 ± 4.55	20.76 ± 10.37	12.91 ± 3.25	19.32 ± 14.63	13.45 ± 7.22
Al	251.88 ± 283.54	111.36 ± 45.29	101.39 ± 94.42	70.87 ± 30.48	189.01 ± 193.37	117.74 ± 91.90
Ca	445.43 ± 662.39	103.91 ± 28.13	259.35 ± 331.08	87.68 ± 26.18	323.72 ± 508.70	117.83 ± 92.16
Fe	220.33 ± 328.96	113.16 ± 28.70	91.06 ± 54.35	128.11 ± 152.16	130.14 ± 116.31	125.30 ± 91.11
K	240.16 ± 167.16	82.83 ± 23.56	339.49 ± 323.49	169.21 ± 84.27	250.49 ± 255.29	106.03 ± 49.22
Mg	46.87 ± 56.22	20.15 ± 9.58	17.67 ± 23.09	11.30 ± 6.60	31.21 ± 35.48	21.94 ± 18.57
Mn	13.71 ± 56.28	3.44 ± 2.25	2.78 ± 2.39	5.92 ± 9.66	3.57 ± 2.96	3.33 ± 2.00
Na	75.82 ± 57.34	31.96 ± 10.19	59.65 ± 27.24	38.27 ± 11.69	68.38 ± 45.93	48.31 ± 25.10
S	444.02 ± 167.26	421.20 ± 164.16	394.62 ± 134.31	476.52 ± 104.85	461.82 ± 144.53	493.21 ± 157.47
Si	726.24 ± 759.68	308.28 ± 115.27	346.62 ± 275.05	198.13 ± 77.35	508.89 ± 530.59	312.47 ± 233.59
Ti	22.40 ± 19.47	15.41 ± 5.37	11.26 ± 7.91	11.14 ± 8.14	16.23 ± 17.31	12.82 ± 9.33
As	1.67 ± 3.74	0.79 ± 0.60	1.27 ± 0.92	1.56 ± 0.63	1.39 ± 1.05	1.25 ± 0.67
Cd	0.74 ± 0.26	0.63 ± 0.11	0.91 ± 0.68	0.74 ± 0.36	0.84 ± 0.57	0.67 ± 0.30
Cr	1.26 ± 0.97	0.78 ± 0.32	1.88 ± 3.02	1.36 ± 0.26	3.38 ± 2.70	2.92 ± 0.81
Cu	6.50 ± 8.27	3.07 ± 0.88	4.92 ± 3.51	4.43 ± 2.43	3.72 ± 2.27	3.72 ± 2.05
Ni	2.34 ± 1.53	2.19 ± 0.75	3.77 ± 1.45	5.20 ± 1.36	10.57 ± 4.60	12.97 ± 3.88
Pb	4.45 ± 12.02	2.19 ± 1.56	3.47 ± 2.32	4.02 ± 1.82	3.29 ± 2.93	3.48 ± 1.99
Se	0.32 ± 0.51	0.19 ± 0.13	0.22 ± 0.16	0.24 ± 0.26	0.25 ± 0.25	0.18 ± 0.13
Sn	1.77 ± 1.39	1.37 ± 1.09	1.68 ± 1.42	1.60 ± 0.99	1.95 ± 1.81	1.22 ± 1.13
V	4.88 ± 3.25	5.03 ± 1.73	8.29 ± 3.18	11.72 ± 2.87	22.72 ± 9.69	28.43 ± 8.23
Zn	33.99 ± 51.74	12.46 ± 4.84	53.59 ± 48.12	33.60 ± 10.43	59.57 ± 44.25	69.11 ± 41.12
Ag	0.59 ± 0.33	0.48 ± 0.02	0.55 ± 0.14	0.48 ± 0.05	0.51 ± 0.10	0.47 ± 0.05
Au	0.77 ± 0.65	0.52 ± 0.37	0.98 ± 0.99	0.66 ± 0.57	0.64 ± 0.57	0.61 ± 0.46
Ba	10.34 ± 9.26	11.22 ± 6.06	6.43 ± 4.71	6.93 ± 4.55	8.55 ± 6.54	14.52 ± 14.59
Bi	0.74 ± 0.61	0.68 ± 0.57	0.75 ± 0.59	0.56 ± 0.40	0.59 ± 0.50	0.40 ± 0.34
Co	0.33 ± 0.62	0.17 ± 0.35	0.36 ± 1.52	0.17 ± 0.29	0.19 ± 0.35	0.16 ± 0.36
Cs	1.48 ± 1.35	1.24 ± 1.40	1.47 ± 1.39	1.81 ± 1.66	1.80 ± 1.64	1.43 ± 1.49
La	4.63 ± 3.67	2.71 ± 2.39	4.55 ± 4.77	3.88 ± 4.02	4.89 ± 4.43	2.54 ± 2.15
Mo	0.24 ± 0.14	0.20 ± 0.01	0.25 ± 0.14	0.20 ± 0.02	0.21 ± 0.04	0.21 ± 0.09
Pd	0.59 ± 0.34	0.50 ± 0.30	0.56 ± 0.20	0.58 ± 0.28	0.57 ± 0.28	0.46 ± 0.13
Pt	3.41 ± 5.26	1.33 ± 1.00	4.49 ± 4.32	3.25 ± 1.58	5.65 ± 4.11	5.95 ± 3.61
Rb	0.18 ± 0.27	0.16 ± 0.16	0.23 ± 0.24	0.33 ± 0.30	0.29 ± 0.30	0.18 ± 0.18
Rh	0.35 ± 0.33	0.27 ± 0.19	0.43 ± 0.46	0.33 ± 0.33	0.34 ± 0.30	0.40 ± 0.50
Sb	1.24 ± 1.30	1.29 ± 1.18	1.70 ± 2.55	1.57 ± 1.58	1.51 ± 1.52	1.40 ± 1.52
Sc	0.40 ± 0.41	0.25 ± 0.06	0.32 ± 0.22	0.32 ± 0.28	0.36 ± 0.35	0.28 ± 0.19
Sr	2.19 ± 2.54	0.99 ± 0.44	1.04 ± 1.06	0.68 ± 0.36	1.60 ± 1.78	1.26 ± 1.08
Te	1.70 ± 1.59	1.13 ± 0.85	1.50 ± 1.44	1.42 ± 1.45	1.14 ± 0.71	1.16 ± 0.80
Th	0.52 ± 0.43	0.42 ± 0.23	0.64 ± 0.60	0.48 ± 0.31	0.57 ± 0.43	0.47 ± 0.29
U	0.54 ± 0.38	0.53 ± 0.41	0.66 ± 0.59	0.49 ± 0.38	0.53 ± 0.47	0.43 ± 0.39
W	5.31 ± 17.78	2.09 ± 2.00	2.73 ± 2.18	2.33 ± 2.14	4.28 ± 4.17	3.38 ± 3.32
Y	0.27 ± 0.25	0.21 ± 0.20	0.26 ± 0.22	0.23 ± 0.17	0.20 ± 0.16	0.20 ± 0.20

Unit: µg/m^3^ for PM_2.5_ and ng/m^3^ for all the other elements; *n* = valid samples included in the final analysis.

**Table 4:** Indoor-Outdoor Ratios for the schools in the three zones.

Zone 1 (*n* = 19)							Zone 2 (*n* = 14)						Zone 3 (*n* = 17)					
Elements	Mean	Median	Stdev	Min	Max	*p*-Value	Mean	Median	Stdev	Min	Max	*p*-Value	Mean	Median	Stdev	Min	Max	*p*-Value
**PM_2.5_**	1.00	0.93	0.59	0.02	2.54	0.453	1.23	0.91	1.15	0.34	5.03	0.541	1.06	0.95	0.61	0.38	2.86	0.719
**Al**	1.23	0.94	1.03	0.09	4.13	0.651	1.43	1.17	1.49	0.50	6.44	0.518	1.11	1.03	0.57	0.30	2.42	0.501
**Ca**	2.61	1.45	2.86	0.15	9.54	0.058	2.31	1.34	2.53	0.69	10.45	**0.034**	1.33	1.15	0.85	0.37	3.26	0.219
**Fe**	1.04	0.86	0.67	0.11	2.71	0.357	0.92	0.93	0.26	0.53	1.50	0.206	1.07	0.97	0.53	0.36	2.46	0.793
**K**	0.95	0.96	0.42	0.08	1.99	0.176	0.89	0.97	0.21	0.41	1.14	**0.043**	1.10	0.96	0.77	0.21	3.32	0.267
**Mg**	1.25	0.96	0.95	0.02	3.45	0.843	3.26	1.06	7.65	0.29	29.63	0.456	1.40	1.14	1.09	0.19	3.98	0.539
**Mn**	1.04	0.95	0.71	0.02	2.63	0.319	1.10	1.10	0.22	0.78	1.63	0.292	1.38	0.98	1.18	0.49	5.58	0.424
**Na**	1.43	1.01	1.20	0.17	5.12	0.359	1.39	1.27	0.49	0.58	2.40	**0.009**	1.27	1.03	0.60	0.64	2.70	0.363
**S**	0.88	1.00	0.34	0.05	1.31	0.112	0.95	0.96	0.19	0.63	1.41	0.168	1.12	0.97	0.63	0.59	3.11	0.878
**Si**	1.23	0.91	1.01	0.12	3.92	0.697	1.37	1.21	1.12	0.52	5.08	0.354	1.06	1.03	0.45	0.32	2.02	0.413
**Ti**	1.09	1.01	0.59	0.11	2.35	0.765	1.05	0.99	0.47	0.43	2.08	0.977	1.36	1.09	1.07	0.30	4.89	0.307
**As**	1.20	0.98	1.15	0.05	5.03	0.853	0.92	0.90	0.57	0.08	2.09	0.212	4.23	0.99	9.23	0.39	28.98	0.535
**Cd**	0.78	0.97	0.28	0.31	1.01	**0.005**	1.12	1.01	0.45	0.93	2.67	0.343	0.98	0.99	0.08	0.77	1.07	0.262
**Cr**	1.29	1.08	0.80	0.07	3.35	0.419	1.09	0.95	0.66	0.38	3.12	0.911	0.98	0.89	0.41	0.58	2.22	**0.052**
**Cu**	0.82	0.90	0.30	0.16	1.25	**0.013**	0.97	1.00	0.29	0.49	1.54	0.342	1.14	0.94	0.77	0.46	3.23	0.715
**Ni**	0.85	0.93	0.39	0.02	1.37	0.088	1.05	0.84	0.89	0.57	4.10	0.922	1.05	0.92	0.61	0.51	3.04	0.074
**Pb**	0.91	0.83	0.73	0.03	2.77	0.246	0.80	0.69	0.43	0.10	1.77	**0.023**	8.65	1.02	15.84	0.07	52.03	0.370
**Se**	1.00	0.98	0.98	0.15	3.15	**0.038**	1.65	1.01	1.79	0.22	7.42	0.437	1.13	1.00	0.97	0.18	4.11	0.417
**Sn**	2.04	1.02	1.82	0.35	5.67	**0.054**	1.80	0.99	1.86	0.39	6.82	0.561	1.80	1.00	1.67	0.28	5.24	0.316
**V**	0.83	0.92	0.35	0.01	1.22	**0.040**	1.03	0.86	0.86	0.51	3.98	0.846	1.05	0.93	0.63	0.49	3.11	0.087
**Zn**	1.06	1.03	0.64	0.04	2.88	0.517	0.99	0.95	0.35	0.55	1.80	0.282	1.08	0.92	0.72	0.59	3.49	0.387
**Ag**	1.04	0.99	0.29	0.68	2.26	0.530	1.00	1.00	0.03	0.93	1.07	0.676	0.98	0.99	0.08	0.77	1.07	0.262
**Au**	1.08	0.97	0.98	0.13	3.82	0.107	1.18	0.99	1.04	0.15	3.63	0.373	2.41	1.02	2.34	0.19	7.91	0.165
**Ba**	0.89	0.74	0.99	0.07	4.76	**0.015**	0.78	0.79	0.54	0.07	2.07	0.059	2.14	1.00	2.41	0.19	7.94	0.988
**Bi**	1.17	0.99	0.84	0.17	3.45	0.416	1.11	0.99	0.86	0.14	2.86	0.240	1.19	0.99	1.00	0.26	3.84	0.797
**Co**	1.93	1.00	2.86	0.01	10.18	0.291	2.94	1.01	4.51	0.28	16.50	0.141	5.12	0.99	12.07	0.77	44.71	0.179
**Cs**	1.17	0.98	1.59	0.26	7.71	0.444	1.71	1.01	1.49	0.30	5.79	0.193	1.30	0.99	1.09	0.49	5.17	0.380
**La**	1.59	0.99	1.59	0.19	4.99	0.583	2.41	1.04	2.31	0.23	7.38	0.145	1.34	0.99	1.22	0.16	4.64	0.490
**Mo**	0.96	0.99	0.10	0.68	1.10	0.116	1.00	1.00	0.03	0.93	1.07	0.676	0.91	0.99	0.19	0.44	1.07	0.102
**Pd**	1.07	0.99	0.72	0.43	4.10	0.807	1.13	1.00	0.49	0.93	2.84	0.354	1.14	0.99	1.03	0.35	5.03	0.978
**Pt**	2.08	1.00	2.13	0.24	6.95	0.462	1.34	0.91	1.65	0.26	6.81	0.475	1.27	0.90	0.95	0.41	3.33	0.275
**Sb**	1.89	1.20	1.60	0.25	5.88	0.085	1.69	1.01	1.61	0.29	5.45	0.419	1.19	0.99	0.76	0.28	3.24	0.991
**Rb**	2.40	0.99	4.22	0.11	15.78	0.875	3.00	1.14	4.28	0.11	14.44	0.561	2.01	0.99	2.37	0.08	7.93	0.590
**Rh**	1.30	0.99	1.13	0.41	5.29	0.415	1.09	1.01	0.55	0.14	2.44	0.986	1.46	1.00	1.68	0.20	7.44	0.913
**Sc**	1.25	0.99	1.23	0.16	5.91	0.862	1.49	1.01	0.97	0.96	3.46	0.082	1.13	0.99	0.62	0.77	3.54	0.426
**Sr**	1.33	1.07	1.02	0.30	4.58	0.267	2.32	1.13	3.62	0.13	14.10	0.357	1.85	1.29	2.42	0.09	10.66	0.758
**Te**	0.73	0.94	0.41	0.22	1.91	**0.006**	1.43	0.99	1.88	0.15	7.91	0.904	2.11	0.99	2.35	0.24	7.88	0.295
**Th**	1.04	1.00	0.58	0.19	3.27	0.368	1.81	1.01	1.61	0.93	6.45	0.084	0.95	0.99	0.21	0.24	1.27	0.480
**U**	1.72	1.00	1.87	0.22	8.69	0.311	1.61	1.01	1.76	0.27	6.98	0.426	2.19	1.02	2.77	0.77	11.32	0.083
**W**	1.61	1.00	1.27	0.68	4.85	**0.045**	0.87	0.98	0.30	0.15	1.03	0.159	0.99	1.01	0.98	0.17	4.50	0.168
**Y**	1.70	1.00	1.23	0.45	4.17	0.153	1.95	1.26	2.05	0.12	7.63	0.502	0.92	0.99	0.80	0.11	3.38	0.164

**Table 5:** Spearman’ Correlation Coefficients at the schools and residences in the three zones.

Zone 1	Al	Ca	Fe	K	Mg	Mn	Na	S	Si	Ti	As	Cd	Cr	Cu	Ni	Pb	Se	Sn	V	Zn
**Al**	1																			
**Ca**	0.91 **	1																		
**Fe**	0.77 **	0.69 **	1																	
**K**	0.46**	0.59**	0.33 **	1																
**Mg**	0.91 **	0.81 **	0.77 **	0.36 **	1															
**Mn**	0.55 **	0.54 **	0.79 **	0.34 **	0.56 **	1														
**Na**	0.81 **	0.85 **	0.67 **	0.70 **	0.71 **	0.57 **	1													
**S**	0.24 **	0.26 **	0.14	0.30 **	0.20 *	0.10	0.24 **	1												
**Si**	0.99 **	0.92 **	0.78 **	0.52 **	0.89 **	0.56 **	0.86 **	0.25 **	1											
**Ti**	0.76 **	0.68 **	0.86 **	0.30 **	0.74 **	0.69 **	0.65 **	0.15	0.76 **	1										
**As**	0.34 **	0.33 **	0.43 **	0.28 **	0.37 **	0.52 **	0.38 **	0.01	0.36 **	0.40 **	1									
**Cd**	0.10	0.03	0.15	0.16	0.05	0.14	0.14	−0.02	0.14	0.22 *	0.11	1								
**Cr**	0.09	0.18 *	0.34 **	0.36 **	0.08	0.41 **	0.27 **	0.14	0.12	0.38 **	0.38 **	0.14	1							
**Cu**	0.31 **	0.35 **	0.59 **	0.43 **	0.27 **	0.62 **	0.47 **	0.04	0.36 **	0.48 **	0.33 **	0.44 **	0.48 **	1						
**Ni**	−0.05	0.00	0.22 *	0.06	−0.04	0.35 **	0.05	0.25 **	−0.03	0.25 **	0.25 **	0.34 **	0.68 **	0.42 **	1					
**Pb**	0.25 **	0.28 **	0.38 **	0.17	0.27 **	0.48 **	0.28 **	−0.06	0.26 **	0.38 **	0.76 **	0.13	0.39 **	0.39 **	0.37 **	1				
**Se**	0.07	0.09	0.09	0.14	0.03	0.05	0.11	0.03	0.09	0.04	0.00	0.38 **	0.07	0.36 **	0.18 *	0.07	1			
**Sn**	0.12	0.08	0.14	0.16	0.13	0.13	0.21 *	−0.03	0.15	0.14	0.18	0.26 **	0.21 *	0.13	0.20 *	0.13	0.23 *	1		
**V**	−0.15	−0.08	0.12	−0.01	−0.15	0.26 **	−0.04	0.28 **	−0.14	0.17	0.16	0.27 **	0.64 **	0.31 **	0.93 **	0.28 **	0.11	0.13	1	
**Zn**	0.23 *	0.39 **	0.34 **	0.55 **	0.14	0.50 **	0.54 **	0.03	0.29 **	0.35 **	0.29 **	0.21 *	0.45 **	0.53 **	0.37 **	0.34 **	0.14	0.12	0.31 **	1
**Zone 2**	**Al**	**Ca**	**Fe**	**K**	**Mg**	**Mn**	**Na**	**S**	**Si**	**Ti**	**As**	**Cd**	**Cr**	**Cu**	**Ni**	**Pb**	**Se**	**Sn**	**V**	**Zn**
**Al**	1																			
**Ca**	0.73 **	1																		
**Fe**	0.73 **	0.48 **	1																	
**K**	0.15	0.38 **	0.15	1																
**Mg**	0.77 **	0.53 **	0.53 **	0.04	1															
**Mn**	0.41 **	0.21 *	0.74 **	0.06	0.33 **	1														
**Na**	0.52 **	0.79 **	0.47 **	0.63 **	0.34 **	0.28 **	1													
**S**	0.16	−0.14	0.26 **	0.08	0.17	0.20 *	−0.09	1												
**Si**	0.91 **	0.87 **	0.63 **	0.31 **	0.64 **	0.27 **	0.70 **	0.00	1											
**Ti**	0.72 **	0.62 **	0.80 **	0.26 **	0.57 **	0.48 **	0.55 **	0.16	0.70 **	1										
**As**	0.17	0.04	0.34 **	0.32 **	0.23 *	0.28 **	0.18 *	0.29 **	0.10	0.32 **	1									
**Cd**	0.00	0.19 *	0.09	0.30 **	−0.09	0.07	0.32 **	−0.12	0.15	0.13	0.22 *	1								
**Cr**	0.16	−0.01	0.25 **	−0.01	0.18 *	0.08	0.03	0.40 **	0.07	0.28 **	0.29 **	−0.02	1							
**Cu**	0.06	0.11	0.25 **	0.45 **	−0.08	0.28 **	0.32 **	0.12	0.14	0.21 *	0.29**	0.23 *	−0.05	1						
**Ni**	−0.14	−0.39 **	0.06	−0.28 **	−0.05	0.06	−0.35 **	0.69 **	−0.28 **	−0.03	0.23 *	−0.10	0.51 **	−0.17	1					
**Pb**	0.13	0.02	0.32 **	0.31 **	0.14	0.39 **	0.13	0.15	0.03	0.26 **	0.74 **	0.16	0.16	0.42 **	0.06	1				
**Se**	−0.06	0.09	0.07	0.21 *	−0.11	−0.02	0.22 *	−0.13	0.06	0.14	0.16	0.50 **	−0.06	0.22 *	−0.16	0.11	1			
**Sn**	0.11	0.17	0.08	0.21 *	0.20 *	0.02	0.25 **	−0.09	0.15	0.15	0.24 **	0.19 *	0.03	0.19 *	−0.13	0.27 **	0.22 *	1		
**V**	−0.13	−0.44 **	0.05	−0.26 **	0.00	0.09	−0.40 **	0.73 **	−0.31 **	−0.06	0.17	−0.15	0.47 **	−0.14	0.94 **	0.06	−0.16	−0.13	1	
**Zn**	0.11	0.33 **	0.23 *	0.69 **	0.00	0.30 **	0.58 **	0.06	0.25 **	0.21 *	0.27 **	0.30 **	0.09	0.41 **	−0.08	0.40 **	0.10	0.15	−0.09	1
**Zone 3**	**Al**	**Ca**	**Fe**	**K**	**Mg**	**Mn**	**Na**	**S**	**Si**	**Ti**	**As**	**Cd**	**Cr**	**Cu**	**Ni**	**Pb**	**Se**	**Sn**	**V**	**Zn**
**Al**	1																			
**Ca**	0.84 **	1																		
**Fe**	0.88 **	0.81 **	1																	
**K**	0.76 **	0.63 **	0.61 **	1																
**Mg**	0.88 **	0.73 **	0.79 **	0.67 **	1															
**Mn**	0.69 **	0.64 **	0.86 **	0.46 **	0.66 **	1														
**Na**	0.72 **	0.73 **	0.79 **	0.64 **	0.61 **	0.77 **	1													
**S**	0.39 **	0.32 **	0.46 **	0.41 **	0.38 **	0.40 **	0.29 **	1												
**Si**	0.96 **	0.89 **	0.93 **	0.72 **	0.82 **	0.74 **	0.77 **	0.44 **	1											
**Ti**	0.91 **	0.84 **	0.95 **	0.65 **	0.83 **	0.78 **	0.73 **	0.44 **	0.94 **	1										
**As**	0.38 **	0.41 **	0.54 **	0.36 **	0.35 **	0.67 **	0.65 **	0.35 **	0.44 **	0.47 **	1									
**Cd**	0.42 **	0.43 **	0.40 **	0.46 **	0.32 **	0.32 **	0.42 **	0.12	0.42 **	0.46 **	0.15	1								
**Cr**	0.44 **	0.43 **	0.52 **	0.41 **	0.37 **	0.41 **	0.39 **	0.61 **	0.51 **	0.50 **	0.15	0.23 *	1							
**Cu**	0.62 **	0.55 **	0.73 **	0.58 **	0.67 **	0.68 **	0.61 **	0.36 **	0.64 **	0.72 **	0.52 **	0.32 **	0.37 **	1						
**Ni**	0.32 **	0.26 **	0.41 **	0.15	0.27 **	0.32 **	0.19 *	0.61 **	0.38 **	0.39 **	0.03	0.06	0.81 **	0.14	1					
**Pb**	0.26 **	0.26 **	0.44 **	0.28 **	0.22 *	0.61 **	0.59 **	0.32 **	0.32 **	0.36 **	0.86 **	0.15	0.19	0.45 **	0.04	1				
**Se**	0.20 *	0.32 **	0.19 *	0.24 *	0.15	0.18	0.21 *	0.16	0.21 *	0.25 **	0.16	0.44 **	0.11	0.17	0.01	0.17	1			
**Sn**	0.25 **	0.27 **	0.25 **	0.29 **	0.21 *	0.28 **	0.33 **	0.18	0.26 **	0.28 **	0.25 **	0.33 **	0.21 *	0.23 *	0.07	0.25 **	0.23 *	1		
**V**	0.30 **	0.24 *	0.39 **	0.12	0.25 **	0.31 **	0.17	0.61 **	0.36 **	0.37 **	0.03	0.03	0.79 **	0.14	0.99 **	0.04	0.00	0.07	1	
**Zn**	0.38 **	0.37 **	0.57 **	0.38 **	0.28 **	0.67 **	0.79 **	0.19 *	0.45 **	0.45 **	0.65 **	0.32 **	0.33 **	0.49 **	0.16	0.73 **	0.12	0.23 *	0.15	1

** Correlation significant at 0.01 level (2 tailed test), * Correlation significant at 0.05 level (2 tailed test), Correlations > 0.6 are shown in bold.

**Table 6:** Varimax Rotated Factor Loading Matrix (Principal Component Analysis) for the elemental concentrations in PM_2.5_ with loadings > 0.5 in bold typeface.

Zone	Z1					Z2						Z3			
Elements	F1	F2	F3	F4	F5	F1	F2	F3	F4	F5	F6	F1	F2	F3	F4
Al	**0.98**	0.07	0.01	0.03	0.03	**0.97**	0.01	−0.03	0.11	0.05	0.02	**0.94**	0.15	0.14	0.09
Ca	**0.9**	0.06	−0.02	0.03	0.15	**0.90**	0.06	−0.22	−0.09	0.03	0.06	**0.79**	0.00	0.14	0.24
Fe	0.41	**0.87**	0.06	0.04	0.13	0.19	0.01	0.06	0.10	**0.96**	−0.01	**0.86**	0.35	0.20	0.03
K	0.2	0.45	−0.04	0.19	0.42	0.00	**0.88**	−0.09	0.08	−0.05	0.37	0.23	0.17	−0.02	**0.86**
Mg	**0.97**	0.14	−0.01	0.04	0.00	**0.89**	0.03	−0.03	0.15	−0.02	0.06	**0.93**	0.13	0.13	0.00
Mn	−0.04	**0.98**	0.04	0.02	0.01	−0.04	−0.01	0.06	0.08	**0.98**	−0.01	**0.71**	**0.52**	0.22	−0.01
Na	**0.65**	0.62	0.04	0.09	0.33	**0.54**	**0.69**	−0.13	0.11	0.21	0.19	**0.64**	**0.62**	0.09	0.22
S	0.07	-0.05	0.44	0.16	−0.35	0.08	−0.01	**0.79**	0.16	0.03	0.00	0.26	0.18	**0.73**	0.12
Si	**0.98**	0.15	0.01	0.03	0.05	**0.95**	0.18	−0.11	0.09	0.06	−0.01	**0.93**	0.17	0.17	0.12
Ti	**0.82**	0.19	0.38	0.00	0.13	**0.57**	0.05	0.09	0.23	0.20	−0.03	**0.76**	0.27	0.09	0.12
As	0.07	0.13	−0.01	**0.97**	0.08	0.21	0.11	0.23	**0.85**	0.08	0.09	0.27	**0.84**	0.04	0.12
Cd	0.31	0.18	0.16	0.19	−0.12	0.25	0.42	−0.10	0.31	0.03	−0.18	0.05	−0.02	−0.10	**0.60**
Cr	0.14	0.18	0.47	−0.01	**0.57**	0.11	0.34	0.23	−0.05	−0.02	**0.76**	0.18	0.27	0.46	**0.69**
Cu	0.09	**0.85**	0.1	0.31	0.2	−0.02	0.29	−0.18	**0.68**	0.24	0.00	**0.55**	0.50	0.08	0.28
Ni	0.07	0.21	**0.93**	0.00	0.13	−0.22	−0.08	**0.91**	−0.04	0.08	0.03	0.17	0.00	**0.95**	−0.01
Pb	0.02	0.04	−0.01	**0.98**	0.06	0.08	0.04	0.05	**0.91**	−0.04	0.14	0.11	**0.91**	0.06	0.09
Se	0.06	0.01	0.03	−0.07	−0.04	0.06	0.04	−0.07	−0.01	−0.02	−0.03	−0.07	0.10	−0.07	−0.06
Sn	0.28	0.43	0.19	−0.07	0.01	0.03	−0.01	−0.11	0.23	0.00	**0.86**	0.49	−0.18	0.07	0.19
V	0.02	0.01	**0.97**	−0.06	0.12	−0.23	−0.15	**0.92**	−0.04	0.01	0.04	0.15	0.00	**0.96**	−0.05
Zn	0.1	0.12	0.11	0.13	**0.78**	0.00	0.93	−0.04	0.15	−0.04	0.00	0.12	**0.82**	0.03	0.02
Eigen Values	6.93	2.78	2.30	1.77	1.12	5.55	2.87	2.54	1.89	1.55	1.25	8.69	2.41	1.99	1.48
% of Variance	34.64	13.90	11.51	8.85	5.62	27.76	14.36	12.72	9.47	7.73	6.25	43.44	12.07	9.96	7.42
Cumulative %	34.64	48.54	60.05	68.90	74.53	27.76	42.12	54.84	64.30	72.03	78.28	43.44	55.51	65.47	72.89
